# Correction of presbyopia using 0.5% pilocarpine eye drops among Indians

**DOI:** 10.6026/973206300200532

**Published:** 2024-05-31

**Authors:** Sharadhi Petkar, MC Chaitra

**Affiliations:** 1 Department of Ophthalmology, Sri Devaraj Urs Medical College, Tamaka Kolar, Karnataka, India; 2 Department of Ophthalmology, Sri Devaraj Urs Medical College, Tamaka Kolar, Karnataka, India

**Keywords:** Presbyopia, pilocarpine, near vision

## Abstract

Pharmacological treatment of presbyopia-spectacle free senescence Therefore, it is of interest to estimate improvement of near vision with 0.5%pilocarpine drops in presbyopic patients. It was a cross-sectional observational study done at tertiary care centre history, comprehensive eye examination, including distant visual acuity distant and near vision, IOP. 1 drop of 0.5%Pilocarpine eye-drops was instilled in both eyes in Patients with full distant vision and impaired near vision, near vision was checked after 2hours. Among 55 participants, 48% were males and 52% were females. Mean age of patients was 44.8 years. Out of 55 patients 58% patients showed improvement of 1 line after instilling drops, 41% patients showed no improvement. Topical 0.5%pilocarpine in treatment of near vision is attractive option for patients and would increase compliance with minimal side effects.

## Background:

Physiological insufficiency of accommodation associated with aging is presbyopia. It is caused ciliary muscles weakening or a loss of elasticity of lens, Presbyopia is anticipated to be increased up to 2.1 billion people globally.[1] Presbyopia presents with problems such as inability to read fine print, need for increased lighting, diplopia, epiphora, headache fatigue or asthenopia, and other tasks, such as threading a needle or seeing fine details on nearby objects. Uncorrected presbyopia directly impacts quality of life of a patient. Hence presbyopic treatment by rest orating of accommodation is the topic of interest. Many treatments option both medical and are being practiced worldwide. Most of the treatments available nowadays are invasive as they are mostly surgical. [1] In recent studies suggest rigidity of lens as a main cause for development presbyopia. Contraction of ciliary muscle, leading to reduce the tension on zonules and further increases thickness of lens, constriction of pupil, and convergence of eyes are 3 processes necessitate the action of accommodation. Although there are many means by which we can restore near vision, access by the patients (most often in the form of reading glasses) is very much bounded in some parts of the world, and it begins at around age of 40 and increases progressively with age. [2] Monovision contact lenses are an alternative option for spectacles; here lens is put on only one eye for near vision. But difference between focusing power of eyes, the depth discrimination, stereopsis, are affected. [3] Hence are in no much use. Multifocal contact lenses are also available but are inconvenient for some patients, who have never worn contact lens. They may also be associated with a risk of ocular surface infections. Hence are cautiously used. Corneal inlay, KAMAR is developed by Acufocus which has a circular aperture & is inserted in the cornea of one eye, pinhole effect is created by the aperture which further increases the depth of focus and improves near vision.[4] Topical treatments which are under study are said to work on various processes of accommodation. Such as miosis which is caused by parasympathetic stimulation, ciliary muscle contraction or softening of lens which in turn restores shape-changing ability of lens. Some disadvantages are bound to happen in each of these methods. Pure parasympathetic drops will result in decrease in diameter of pupil and myopic shift, which compromises distant vision, many adverse reactions have also been noted using these drops. Pilocarpine eye drops works by contracting ciliary body and inturn stimulating accomodation, miosis caused by this drug also increases depth of focus, which increases the depth of focus. Pilolocarpine HCl Ophthalmic Solution 1.25% is a U.S. FDA Approved Agent for Presbyopia. [5] Different drug combinations have been tried & used in pharmacological treatment of presbyopia in recent years. Pharmacological treatment, gives us a benefit of having a spectacle-free condition with a less risk of ocular complications, compared to surgery. [6] Therefore, it is of interest to estimate improvement of near vision with 0.5%pilocarpine drops in presbyopic patients.

## Materials and Methods:

## Study design:

A Cross sectional observational study.

## Source of data:

Presbyopic patients who visit Ophthalmology OPD at R. L Jalappa Hospital, Kolar attached to Sri Devaraj Urs Medical College, Tamaka, and Kolar, Karnataka, India.

## Study duration:

6 months

## Inclusion criteria:

All Patients aged 41-65 years of age who are emmetropic for distant vision in both eyes (6/6 by Snellen's Chart) but have difficulty in near vision only. (Jaeger's Chart < N6) [Who have spectacle dependent near vision and spectacle independent far vision].

## Exclusion criteria:

[1] Patients who underwent refractive surgeries.

[2] Pseudophakics

[3] Patients with other ocular comorbidities like dry eye, glaucoma, retinal problems.

After obtaining an informed consent, demographic details were noted. followed by a comprehensive eye examination which include distant visual acuity is checked by Snellen's chart and near vision by Jaeger's chart, extra-ocular movements, anterior segment evaluation with the help of slit lamp and fundus examination by direct & indirect ophthalmoscopy. Intra ocular pressure by non-contact tonometer was performed for all patients before instilling pilocarpine drops. Patients with full distant vision and impaired near vision was taken up for study. One drop of 0.5% Pilocarpine eye drops are instilled to both eyes of patient and patients near vision is checked after 2 hours of instillation of drops. Improvement of near vision after instillation of drops is noted. Data will be coded and entered into excel sheet. All quantitative measures will be presented by mean and Standard Deviation. Chi-square will be the test of significance. Continuous data will be represented as mean and standard deviation. Paired t test will be the test of significance to identify the mean difference between paired data. P-value < 0.05 will be considered as statistically significant.

## Results:

55 participants were included among them 48% were males and 52% were females (Figure 1). Mean age of patients was 44.8 years (Figure 2). Out of 55 patients 58% patients showed improvement of 1 line after instilling drops, 41% patients showed no improvement (Figure 3). In a sample of 55 presbyopic patients, pilocarpine instillation demonstrated a statistically significant improvement in near vision (Table 2). The mean difference (d) was -4/3 LogMAR units (SD = 1), resulting in a paired t-test statistic (t) of -9.888 (df = 54, p <0.05 p<0.05). These findings suggest a notable enhancement in near vision following pilocarpine treatment.

## Discussion:

Presbyopia treatment by pharmacological method is very fascinating option for patients. In this observational study, we investigated the impact of 0.5% pilocarpine on individuals with presbyopia. The application of 0.5% pilocarpine demonstrated a significant increase in near vision, indicative of positive clinical efficacy of pilocarpine. Participants of age group of 40-45 showed subjective satisfaction of increased comfort and improved visual acuity at near distances with no side effects. Positive feedback suggests acceptability if 0.5% pilocarpine in treatment of early presbyopia. Many drug combinations are under trail to reduce side effects and increase efficacy of currently available drugs. In our study we used minimal concentration of pilocarpine and observed one line improvement which was also observed in phase 2 trials by Price *et al.* [7] Maximum improvement was seen in age group of 40-50 years of age which shows its significance in treating early presbyopia. Only 2 among 55 subjects had brow ache following instillation (Table 1) which is also seen in >5% population in study conducted by Abdelkader *et al.* [8]. This proves low concentration of drug may show less adverse effects or increased comfort. In our study Improvement of near vision was just one line, which proves the fact that optimal concentration of pilocarpine should be 1% or more for significant effect on presbyopia. [9, 10] Thus, we have educated patients about the availability of eye drops for treating presbyopia where no patient in our study was aware about.

## Conclusion:

Presbyopia is presently being treated with a variety of newer pharmacological treatments, with encouraging positive results. Recent literature search shows many studies being done using different combinations of drugs for treating presbyopia. But with 0.5% pilocarpine for presbyopia, was done for the first time ever and there is one line improvement which can be used for patients with early presbyopia with very minimal side effects.

## Financial support and sponsorship:

Nil.

## Figures and Tables

**Figure 1:**
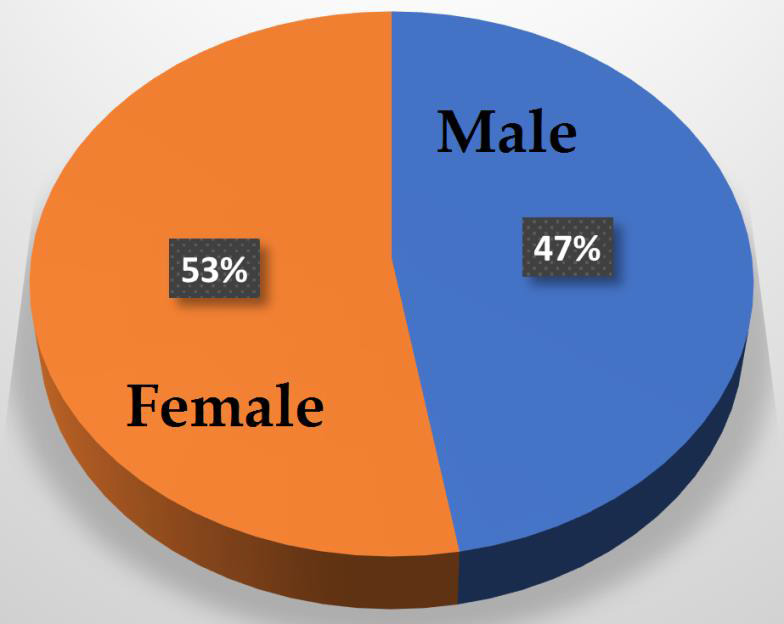
Distribution of males & females in the sample

**Figure 2:**
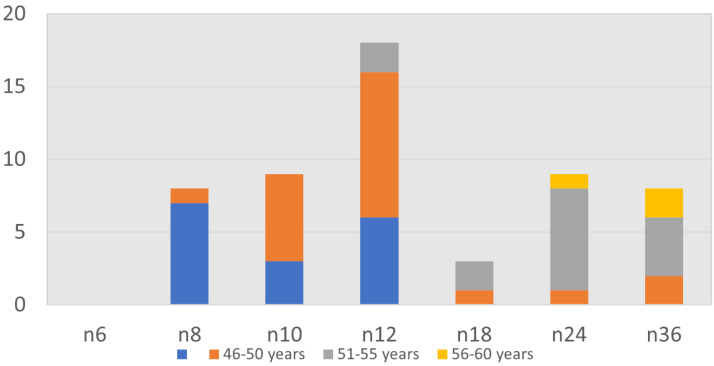
Age wise distribution of near vision

**Figure 3:**
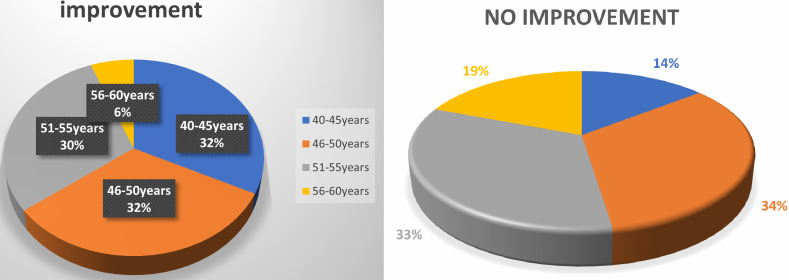
Improvement and no improvement with pilocarpine

**Table 1:** Table1:Side effects after pilocarpine

**Side effects After pilocarpine**	**Number of subjects**
Brow ache	2

**Table 2:** Age wise distribution of near vision

**Side effects After pilocarpine**	**Number of subjects**
**Brow ache**	**2**
**AGE GROUP**	**IMPROVEMENT OF NEAR VISION**
40-45	32%
46-50	32%
51-55	30%
56-60	6%
